# Vaccine Refusal: A Preliminary Interdisciplinary Investigation

**DOI:** 10.3389/fpubh.2022.917929

**Published:** 2022-07-22

**Authors:** Linda A. W. Brakel, Gordon R. Foxall

**Affiliations:** ^1^Department of Psychiatry/Philosophy, University of Michigan, Ann Arbor, MI, United States; ^2^Cardiff Business School, Cardiff University, Cardiff, United Kingdom; ^3^School of Business, Reykjavík University, Reykjavík, Iceland

**Keywords:** akrasia, addiction, future-discounting, informational-reinforcement, dual-processes, rationalities, non-knowing, identity

## Abstract

Many people who generally receive standard recommended inoculations refuse to partake of COVID-19 vaccines, preventatives that are effective, safe, and life-saving amidst the current pandemic. Our quest is to understand this puzzling and dangerous phenomenon, as it exists among US and UK citizens, whom in other respects would be regarded as quite regular. We will discuss Vaccine Refusal compared with two better understood phenomena: addiction, and akrasia, along with the related matters of human action, intention, agency, will, and identity. Vaccine Refusal, we will argue, appears to be rewarded by “informational reinforcement” leading to heightened arousal, along with increases in self-esteem resulting from “bucking the trend,” asserting one's “superior” understanding, and “tribal identity” in acting against social norms. These factors provide an overall reward amounting to satisfaction that outweighs the well-known consequences of COVID-19 infections. Our investigations will also lead us to a pair of epistemological hypotheses about two subtypes of the Vaccine Refusers under consideration here.

## Introduction

How is it that so many people refuse to partake of a COVID-19 vaccine, a preventative that is effective, safe, and life-saving (1)—this amidst a pandemic the likes of which the world has not seen for a century? This is the question which launched and continues to animate this interdisciplinary project. Certainly, for too many in the non-“first world” world, vaccine protection is not available. This represents a different set of problems, both economic and moral—problems not addressed here. Rather, given clear unequivocal data demonstrating that serious illness, hospitalizations, and deaths are far lower for those who are vaccinated, we will try to understand how vaccine refusal^1^ can remain a serious deterrent to better management of the pandemic.

One possibility is that the ameliorative vaccine statistics are not readily accessible. However, that is unlikely as these data are widely circulated in the United States,^2^ the United Kingdom, and the^1^,^2^ European Union. Now it can be the case that this information arrives discounted, distorted, denied, which itself is likely part of the vaccine refusal story. But even so, as New York Times Columnist and Nobel Prize economist Paul Krugman wrote (New York Times, January 21, 2022) (3): “Certainly most Americans, even if they haven't developed COVID themselves, know people who have gotten seriously ill or died…,” adding that “What makes…this especially demoralizing is that 2021 began with the hope that miraculous vaccines would end the pandemic.” He continues: “Despite the effectiveness of the vaccines in preventing serious illness, that didn't happen…individuals fail to get vaccinated…” Putting it more bluntly, most know persons who have become very ill or who have died, and know too that many of those need not have suffered, were they to have gotten vaccinated.

Before continuing, we should make clear just what sorts of Vaccine Refusers are under exploration for our project, and which are not. Those people who are refractory to any expert advice—specifically those who repel any and all “elite” recommendations, including advice from established scientific and public health agencies—along with those who are simply unintelligent non-thinkers, comprise two groups which will not be taken up here. Further, people who in general refuse all vaccines, and have done so for years, will not be considered. These so called “anti-vaxxers” represent a different population from those who have accepted and continue to receive various vaccines, but refuse all COVID-19 vaccinations. Also, not addressed are people who are vaccine-hesitant regarding the COVID-19 injections. While those in the vaccine-hesitant group may seem not easily distinguished from Vaccine Refusers, we aver that there is a significant distinction. Whereas the vaccine-hesitant hold beliefs about COVID-19 vaccinations that can be changed as evidence accrues, this is not the case for the Vaccine Refusers investigated here. To state it plainly, the Vaccine Refusers investigated here are rigidly committed to refusing any COVID-19 vaccine.

Now that the type of Vaccine Refusers under consideration has been specified, we will compare this sort of Vaccine Refusal with two better understood phenomena: addiction, and akrasia, along with the related matters of human action, intention, agency, will, and identity. These explorations will also lead us, finally, to a pair of epistemological hypotheses about two types of Vaccine Refusers. While this interdisciplinary project is unlikely to provide totally satisfying answers to the original (and vexing) question regarding COVID-19 Vaccine Refusal, we hope to have offered more information, geared toward the general good.

To start, because they cross cut throughout this entire exploratory endeavor, two intertwined issues will be taken up first: (1) rationality and (2) dual process mentation. In particular we will examine three forms of rationality—Economic, Philosophical/Psychological, and Biological Rationality; and two sets of mental modalities—System One/ Primary Process and System Two/Secondary Process.

## Three Types of Rationality

In an instructive and useful typology, Kacelnik (4) distinguishes three rationalities that are helpful in discussing the nature of Vaccine Refusal, albeit with some modification and extension given the current context. In what he terms “PP-rationality,” psychologists and philosophers emphasize the process in which decisions are made, asking whether it is in accord with principles of rational thinking. Economists, by contrast, stress the consistency of choice behaviors in the course of decision making, regardless of the particular goals reached or the processes employed (“E-rationality”). For biologists, however, the achievement of fitness superceding that of conspecifics is the criterion of rational behavior (“B-rationality”). None of these, however, he points out, is sufficient to capture the idea of rationality, and we have, indeed found it necessary to extend his classification.

Economic Rationality, for instance, must take account of what we refer to as Economic-Psychological Rationality which extends the basis of utility and thereby elucidates the motivation of Vaccine Refusers. Philosophical/Psychological Rationality, often juxtaposed simply with “irrationality,” must, it is argued here, take account of the possibility of “a-rationality.” Finally, Biological Rationality—founded on the understanding that living things maximize biological fitness, both selective reproductive fitness success and the related inclusive fitness—requires attention owing to the apparent blatant abrogation of biological viability in the Vaccine Refusal group, and with respect to a broader societal notion of extended inclusive fitness. With these considerations in mind, we now examine each in turn and reach a judgment of what is missing insofar as rationality is a consideration in the avoidance of COVID-19 vaccinations.

### Economic Rationality

#### The Nature of E-Rationality

Economics assumes that a rational actor, such as a consumer, maximizes utility (defined roughly as the amount of satisfaction he/she gains from owning and consuming commodities). Moreover, E-Rationality based on the assumption that an economic actor will be consistent assumes_the transitivity of preferences: a consumer who prefers product x to product y and y to z, must necessarily choose x rather than z. This pattern of responses displays transitivity; to deviate from it by selecting say x over y, y over z but z over *x*, shows intransitive preference, inconsistency and therefore lacks Economic Rationality [e.g., Rubinstein (5)].

There are many instances where this preference axiom is not met in practice [See Houston (6)]. For one thing, if the consumer's state alters, he/she may display apparently economically-irrational behavior without our questioning her consistency. As Kacelnik (4) (p. 92) points out, his preferring lamb to ice cream at 8 p.m., ice cream to coffee at 9 p.m., but his nevertheless choosing coffee over lamb at 9.30 seems to contravene the principle that acts of consumption display transitivity. However, in practice, such a pattern of behavior scarcely attracts the criticism that it lacks consistency since there has been a change in the individual's state in the course of this sequences of choices. In standard neoclassical formulations of consumer rationality, for instance, there is a rather rigid assumption that tastes do not change through time. (Later formulations of economic behavior assume, less rigidly, that only broad categories of consumption objects remain unchanged through time.) But those human consumers who discount the future hyperbolically display preference reversal in the course of intertemporal behavior. They often set out to accept a larger later reward, but just as a smaller less delayed substitute becomes available, they switch to it. I may be resolute after breakfast in planning to avoid a palatable but unhealthy food at lunchtime, but comes the midday break it is easy to give in. This is economically irrational only if one has a rather rigid view of the consistency required of a rational economic actor. But I may determine that I will save over 2 years for a Bang and Olufsen sound system, only to buy an inferior product after 6 months just because I now have the money for it. Even less rigidly, after a year of saving, I may spend the sound system money that has accumulated on a vacation, a choice of a totally disparate consumption category. Even this is not irrational if my tastes can be sufficiently broadly conceived as entertainment.

However, if I spend a large sum of money on a weight loss program, only to indulge frequently in forbidden foods thereafter, my behavior is by all accounts economically irrational, because I am undermining the utility to be gained from my expenditure by incurring further pecuniary and non-pecuniary costs that act against that utility being realized. Which, if any, of these behaviors characterizes the Vaccine Refuser?

#### Taking Note of Economic-Psychological Rationality

E-Rationality, that attributed on the basis of economic reasoning, differs from that found in economic psychology which emphasizes both the economic consistency of behavior and its modification by the thinking and feelings of individuals. Economists generally understand utility in terms of the objective attributes provided by products and services; this facilitates economic calculations and comparisons of commodity sets that make a variety of attributes available. However, Foxall (7, 8) has proposed a broader understanding of utility in terms of the kinds of reinforcement a consumer achieves through consumption. We might refer to this as Economic/Psychological Rationality (EP-Rationality). Utilitarian reinforcement approximates the economist's conception of utility, namely the functional benefits of acquiring and using economic goods. In addition, however, there is evidence that humans regulate the rate of their behavior in line with the informational consequences it has previously produced. Posting scores or other measures of performance affects the frequency of behavior, for instance, especially in social situations. While utilitarian reinforcement is mediated by the product whose use supplies functional rewards, informational reinforcement is socially mediated; the rewards it confers take the form of social esteem, identity, honor, regard. Ultimately, it inheres in the self-esteem the actor feels as a result of behaving in a particular manner.

Crucially therefore, the consumer's utility function comprises not only the utilitarian reinforcement specified by the economist but also the informational reinforcement recognized by economic psychology. What the consumer actually maximizes is a combination of utilitarian and informational reinforcement [See Oliveira-Castro and colleagues (9–11)]. Since informational reinforcement is a subjective reward, something ultimately conferred by oneself on oneself, and because it may vary with the state and current circumstances of the individual, it is a frequent source of changes in one's utility function and its effect may be to render one's behavior apparently irrational to an onlooker. This may be the reason why human choices often are modified with the passage of time, why preference reversal is commonly observed in successive situations that offer differing levels of reward [See Ainslie (12), Foxall (13)].

### Philosophical/Psychological Rationality

#### The Nature of Philosophical/Psychological Rationality

The criterion of rational vs. non-rational beliefs is the manner in which they are arrived at, rather than their contents or the behaviors to which they lead. Kacelnik (4) is clear that what qualifies as a rational belief based on the information available at the time of its inception, may turn out to be non-rational if new understandings arise. Hence, belief in the geocentric nature of the solar system was rational in an era which relied on naïve observation of the sun and the earth but yielded to a heliocentric view with the enhanced sophistication of physical instrumentation. Scientific and technological advance will yet entail similar modifications of beliefs which today are entirely rational. The tentative nature of belief is an important take-home message from consideration of the nature of PP-Rationality.

Nonetheless, irrationality is possible in the case of individuals who avoid information which is currently available, who deliberately or purposefully take no note of what is known and what is likely, especially if the potential sources of the knowledge they might take on board and allow to influence their behavior are systematic, scientific and based on generally accepted methodologies that work in other contexts such as experimental analysis. However, we argue that most in the Vaccine Refusal group we consider here, are not irrational in this sense: a nuanced understanding of their behavior requires a more subtle appreciation of the nature of information and belief. Vaccine Refusers often seek information and draw conclusions in a manner similar to others, a quasi-scientific methodology. So, a criticism of Vaccine Refusers founded on the idea that they are not following P–P Rationality, does not go far enough. One needs to appreciate the kind of mentation within which they are operating.

#### Taking Note of a-Rationality

Economic/Psychological Rationality suggests that an individual can so configure their behavioral responses that they yield a degree of personal reward based on the ability to feel self-esteem according to the exercise of one's discretion. This may range from denial of the reasons customarily given for what is presented as rational behavior to outright defiance of the conventional wisdom for regulating his/her behavior. If such action is based on a radical and groundless rejection of the reasons for sensible conduct, it may be referred to as irrational, but this pathological response is rare. Persons who in general function normally within society may well assume a subjective interpretation of the facts behind the advocacy of a specific course of action. But such deviation from social norms and mores is seldom completely groundless; rarely do individuals base their actions on no intellectual or moral justification whatever. The bases of reasoning that justifies the deviant activity may be erroneous but such reasonings are not absent. Although such a*-*rational behavior is socially deviant, it is not performed without any justification whatever. The problem is that, although the kinds of belief that may plausibly justify action is reasonable to the neurotic, these beliefs are not reality-tested.

### Biological Rationality

#### The Nature of Biological Rationality

Fitness assumes the role in biology that is occupied by utility in economics. The behavior of individuals is the result of the genetic material that has been provided in the course of evolution by natural selection. Rationality is, therefore, not to be sought in the conduct of the agent but in the biological procedures that have emerged from the evolution of biologically rational mechanisms. It is these mechanisms that determine whether the behavior of a single agent is rational; as Hurley and Nudds [(14), p. 22] summarize it, “A *B-rational* individual is one whose behavior maximizes its inclusive and selective reproductive fitness across a set of evolutionarily relevant circumstances.”

#### Taking Note of Extended Inclusive Fitness

Vaccine Refusing persons clearly evince biological irrationality by reducing their own selective reproductive fitness – since the chance of a long (reproductive) life is mitigated by rejection of a tried and tested means of prolonging and enhancing life. Likewise, their inclusive fitness status is compromised, as they are unlikely to be able to fulfill the social requirements of ensuring that kin are capable of successful reproductive cycles themselves. This is more problematic in a modern complex society in which social interactions are multifarious and involved.

Moving away from Kacelnik's (4) idea of strict B-Rationality, it is noted, that often humans act altruistically toward non-kin, e.g., adoptive parents, aid to complete strangers. This can be designated as “extended inclusive fitness,” noting that Vaccine Refusal represents ongoing harm, rather than altruistic regard, toward all others who Vaccine Refusers encounter, clearly damaging their own extended inclusive fitness in the process. Taking this into consideration, it is clear that individuals who fail to maintain their own health and wellbeing are likely to be less economically productive (including paying taxes, being generous to others) and hence less socially productive in enhancing the fitness of people generally.

## Two Sets of Mental Modalities

### Primary and Secondary Process Mentation

Freud (15) in 1900 in *The Interpretation of Dreams* presented a clear outline of two different formal modes of mentation he had observed. ^3^ The more basic type—developing earlier and quite obvious in young children, but also in the everyday dreams and daydreams of normal adults, as well as in persons under stress and those with neurotic problems—Freud termed the Primary Processes. He proposed that primary process type mentation was present as unconscious background in much of adult human behavior, but that the more familiar secondary processes were uppermost in the conscious “rational” operations of alert and wakeful adults.^4^

Unlike the Secondary Processes, which (among other functions) attempt to evaluate incoming information with respect to discerning what is real and true, the Primary Processes operate prior to considerations of what is true and what is false. Therefore, they are properly considered a-rational rather than irrational in that they lack, rather than violate, the principles of everyday logic [(16), p. 58]. The Primary Processes operate such that opposites are not mutually exclusive, contradictions are tolerated, and reality testing, including evidence-based reasoning, is not employed [(16), p. 58]. Further, contextual time, both past and future, are not registered as such. Thus, when Primary Process mentation predominates one can exist in what Brakel [(17), p. 131] has termed “the unexamined present.”

Equally important, the Primary Processes employ associative, rather than causally based connections. This has significant implications for categorizations. Primary Process-based categories can be regarded as more a-rational than rational in that they predicated on contiguity in time and space, and superficial resemblance of small attributes, including part-for-whole feature similarities. Secondary process categorizations, in contrast, aim for more central, essential, or causally-based etiologic resemblances among category members [(16), pp. 58–59].

Finally, the Primary Processes are faster and closer to drives, instincts, and affects than are the Secondary Processes (15). Indeed, the Secondary Processes strive for solutions that are largely affect-independent, sometimes overriding emotions, but always reality-tested, evaluating evidence. And unlike the impulse-linked, quick, reactive Primary Processes, the Secondary Processes are more deliberative and require more psychological work and energy.

### Dual Processes—System One and System Two

Many of the same contrasts just outlined between the Primary and Secondary Processes are reflected also in differences between so called “System One” and “System Two” modes of cognition. These “dual processes” have been posited and then empirically demonstrated by modern cognitive psychologists, perhaps best known of which are Tversky and Kahneman. Working together, sometimes with other researchers, and sometimes solo, Tversky and Kahneman have provided a large body of empirical work all supporting System One and System Two, as two mentation types.^5^ [See for example: Kahneman et al. (18), Tversky (19), and Kahneman (20)].

System One operates with speed; quick reactions take precedence over logical, considered deliberations. Even complex situations are addressed rapidly, with “solutions” short circuiting the arduous mental energy System Two-type mentation requires. Like the Primary Processes, the System One modes of cognition develop earlier, and are similarly tied to impulses and emotions. It is then not surprising that System One responses can seem (and be) automatic. Also similar to Primary Process mentation, System One operates with associative rather than causative connections. The later developing System Two favors reasoning, strives for rationality, and obeys the rules of everyday logic. Interestingly, both System Two and the Secondary Processes, function more than occasionally to slow, modulate, and sometimes even directly inhibit the System One/Primary Process outputs.

To get a better appreciation of System One type operations, here are some of the specific processes most often characterized as such (18–20): (a) Representability and availability are over counted. Thus, unusual and intense circumstances are viewed as more likely to occur, and represented with greater frequency than is accurate. (b) Recency and final effects (sometimes called framing) are more prominent. (c) Risk and loss aversion predominate over gain possibilities. (d) Transitivity is not respected. (e) Similarity can be based on inessential attributes.

Interestingly, depending on how one defines one's goals, operations with System One predominating can sometimes provide more effective outcomes than can System Two. For example, if increased group cohesion is a desirable effect, singular focus on a shared intense circumstance may produce and accelerate this outcome.^6^

Having discussed the preliminary tools necessary for our investigation—the three forms of rationality and the two types of mentation— basic questions about the nature of Vaccine Refusal will now be addressed the first of which follows in the next section just below.

## Is COVID-19 Vaccine Refusal Any Sort of Addiction?

The short answer to this section's title question is “No.” On our view, COVID-19 Vaccine Refusal is not an addiction. However, along with important central differences, there are features in common between Vaccine Refusal and Addiction. So, after defining Addiction, the similarities and then the salient differences between Vaccine Refusal and Addiction will be taken up.

### Addiction—A Working Definition

Addiction defies simple definition.^7^ For Koob et al. [(24), p. 4], “drug addiction is a disease and, more precisely, a *chronic* relapsing disease” (emphasis in original). More particularly, the authors elaborate that in the context of substance consumption, addiction is “a *chronic* relapsing disorder characterized by compulsive drug seeking, a loss of control in limiting intake, and emergence of a negative emotional state when access to the drug is prevented” (p. 24). Foxall [(13), p. 181] proposes, more broadly, that addiction is “a mode of consumption marked by steep temporal discounting and preference reversal, involving the pursuit of a substance or behavior pattern to the point of economic irrationality, where it fundamentally disrupts the individual's lifestyle, and is sustained by neurophysiological excess, “midbrain mutiny,” as Ross et al. (25) characterize it.”

Addiction is more a process than an event. It is marked by acute initial positive reinforcement, rewarded by strong feelings of pleasure and arousal, i.e., definitive liking, followed by tolerance and, on cessation of the administration of the substance or the pattern of behavior, withdrawal symptoms that manifest in strong negative reinforcement, and the search for a diminishingly (positively) reinforcing object [see, e.g., Koob (26)]. Despite this constant search which can take over the life of the addict, the substance or practice is no longer liked or enjoyed in itself. Liking has given way to wanting,^8^ and wanting or incentive salience is exacerbated by the discriminative stimuli and motivating operations that compose the consumer behavior setting, and which have been associated through classical conditioning with the substance or behavior itself. This wanting, and as time goes on, *craving* the object of addiction can lead to such breakdowns in normal social functioning as loss of a partner, loss of a home, aloneness and loneliness. These all play a part in the delineation of addiction.

What may link the various instances of addiction, be it to substance or behavior, appears to be a change from the tonic rate of action potentials in dopaminergic cells in the mesolimbic pathway, which is associated with an alertness to opportunities for reward, to a phasic rate when incentives of large-scale learned salience become real possibilities. The admittedly very small proportion of the population who display gambling addiction for instance “learn that if the organism whose consumption they guide gambles extremely frequently, this permanently elevates tonic dopamine, disables GABAergic inhibitory signals, and thereby turns the slot machine or home computer into an easily self-operated phasic dopamine pump” [Ross (27), p. 60; see also Ross et. al. (25)].

### Vaccine Refusal and Addiction: Features in Common

Since late 2020, it has been well established (1)^9^ that the approved COVID-19 vaccines^10^ reduce severe disease, hospitalizations, and deaths, while having a very favorable safety profile. In particular adverse side effects from the vaccine are overwhelmingly benign, and even those that are more serious^11^ are not only extremely rare (occurring more often in COVID-19 infections than as vaccine side-effects), but are highly treatable, and very short lived, remitting with no lingering ill effects. These happy outcomes are not so for COVID-19 infections. Although the infection can be asymptomatic or mild, it is equally possible that COVID-19 can be deadly, rendering one severely ill, requiring hospitalization, perhaps even an ICU stay in order to survive. Moreover, and relating directly to the issue at hand regarding addiction (as will be obvious immediately below), a COVID-19 infection (even an asymptomatic or mild one) can occasion “long-COVID,” which includes a range of symptoms compromising one's good health for weeks, months, or even years.

As is true for people with addictions, people who are COVID-19 Vaccine Refusers are discounting the future. They are opting for some sort of Short-term Small Reward (SSR) over a clearly better health picture in the future—this, a Long-term Large Reward (LLR). Filling out the content of the equation such that Short-term Small Rewards are preferred over Long-term Large Reward SR (SSR > LLR) is easy and routine for those with substance abuse addictions, especially at the start. For example, the SSR of a cigarette for a smoker, consists of a unique state of feeling of both increased focus and greater calm; while the long-term damages to the lungs and cardiovascular system seem far away, and not immediately perceived. The general cellular carcinogenic effects of smoking are regarded as even more distant, not even perceivable. Thus, the Long-term Large Reward (LLR) of better health is discounted.

But how does one fill in the content for the COVID-19 Vaccine Refuser? The LLR of better health is clear, but what factors comprise the Short-term Small Rewards, the SSRs that are valued over and above good health? To get to some satisfying answers let us begin more generally, actually in two steps. First, following Foxall's (7) work *Addiction as Consumer Choice*, posit at the outset that one can better comprehend aspects of addiction by classifying addictive behavior as a particular mode of consumer behavior. The second step involves understanding the role of two disparate sorts of reinforcements as reward sources contributing to consumer behavior.^12^ Foxall [(7), p. 74] describes these two as follows: “…*utilitarian*, which refers to the functional benefits of purchasing, owning, and using a product or service; and *informational*, which consists in the social consequences of these activities, the social honor, prestige, and status that others confer upon the owners and consumers of certain economic goods and services.” Clearly for the Vaccine Refuser, the utilitarian reinforcements are sparse, while the informational ones are rich, varied, and plentiful. Indeed, this along with SSR > LLR, seems a feature that Vaccine Refusal shares with Substance Addictions.

Now, to address the above vital question of what constitutes SSRs for Vaccine Refusers, the specific reinforcement rewards (of both reinforcement types) potentially promoting Vaccine Refusal will be outlined. As far as utilitarian reinforcements, functionally there is the non-trivial matter of convenience. No matter that vaccines are free, widely available, and fairly readily accessed, it is easier to *not* obtain the vaccine, especially as two to three (or more) injections are needed to be fully protected. One must have transportation to get to a vaccination site, perhaps arrange for child care, and maybe miss some work time. Also, there are some slight discomforts associated with the vaccine—the necessity of wearing a mask, the minimal jab of the injection itself, and the possibility of mild side effects including pain at the injection site, headache, mild fatigue etc. over the next day or two. In short, the utilitarian reinforcements, amounting to a short-term small reward (SSR), consist largely of the avoidance of these minor pains.

The informational reinforcements for Vaccine Refusal are more profound. They include ready membership in a political/social/tribal group (certainly so within the US) that is stridently vocal about “personal freedom.” Relatedly, the leaders of this group, consisting of politicians, media journalists, and celebrities, all of whom offer approbation for Vaccine Refusal, accelerate the informational reinforcement and with it the perceived short-term award. The belong-to-a-tribe identity, the praise from within the group, the high-minded notion of participating in a fight for personal liberty—these clearly constitute strong short-term rewards—rewards that are chosen over the long-term large reward of future good health. But it is more questionable whether this set of rewards is be considered only “short and small”.

Continuing with similarities between addiction and Vaccine Refusal, there is the phenomenon of increased seeking of the addictive reward whenever materials and entities *associated* with one's addiction are present.^13^ Under these conditions, addicts, even reformed addicts, experience increased pressure to seek their addictive drug. (See for example, Berridge and Robinson, [(28), p. 3]; Perry, [(29), pp. 4636–7]). For Vaccine Refusers, the increased (almost addictive) seeking of vaccine misinformation, particularly when it originates from group leaders associated with Vaccine Refusal, can be considered analogous behavior. This is likewise true of renewed hardening of the Vaccine Refusal position whenever it is challenged, for the challengers represent persons associated (in the negative) with Vaccine Refusal. The misinformation about vaccines usually concerns their supposed ineffectiveness, and worse, their putative danger.

### Vaccine Refusal and Addiction: Distinguishing Features

There are very important ways in which Vaccine Refusal is different from any Addiction. For many with addiction,^14^ along with future discounting and the characteristic repeated self-damaging behavior, there is another central phenomenon—preference switching. (Foxall [(7), p. 5].) Preference switching (or preference reversal) is seen in those with addiction as follows (Foxall [(7), p. 45]):

…an initial preference for the LLR [i.e., good health]…abruptly morphs into a preference for the SSR [the addictive substance] just as it becomes available. It is also true that [addicted] individuals switch preference in the opposite direction, as when a heavy drinker chooses sobriety…the delayed reward of better health…over the immediate pleasures of alcohol.

At least for the group of Vaccine Refusers of concern, neither of these preference reversals takes place. Although there are some in this group who do believe that there could be negative consequences in not getting vaccinated—the most important of which would be getting seriously ill—even for these people there are other SSRs and LLRs that outweigh the important future good health LLR. For instance, there are SSR and LLR combinations in tribal-type belonging, praise from and identification with group leaders, and dedicated misinformation seeking. These all provide an initial and stable preference for Vaccine Refusal. The other (perhaps larger) faction of our Vaccine Refusal group does not perceive COVID-19 vaccination as offering the LLR of future good health at all! Hence, Vaccine Refusal is not at all challenged. The SSR of not being vaccinated is the first preference and remains so, and strengthens (enlarges and lengthens) with other reward combinations. There is one exception, seen in both subgroups: Preference reversal toward getting vaccinated has occurred (not infrequently) after the Vaccine Refuser him/herself becomes gravely ill with COVID-19, or if this befalls a beloved other. Often this preference switch comes too late.

There is another almost definitional phenomenon experienced frequently during addiction, but not at all with Vaccine Refusal. As an addiction continues over time, the sufferer increasingly wants the addictive substance more, even as he/she likes it less. This finding owes to the pioneering work of Berridge et al. (31) [see also Robinson and Berridge (32)] who put forth the now increasingly accepted “incentive-sensitization theory of addiction.” Here is the basic idea as summarized by Berridge and Robinson [(28), p. 670]:

…the brain circuitry that mediates the psychological process of ‘wanting' a particular reward is dissociable from circuitry that mediates the degree to which it is ‘liked'…The incentive-sensitization theory posits the essence of drug addiction to be excessive amplification specifically of psychological ‘wanting', especially triggered by cues, without necessarily an amplification of ‘liking'.

With Vaccine Refusal there appears to be a steady state of equivalence between “liking” and “wanting” in that Vaccine Refusers want to be unvaccinated and they like choosing not to get the COVID-19 vaccines.^15^

This subsection will close with the assertion that since the distinguishing features of addiction are missing for Vaccine Refusers, Vaccine Refusal is essentially different from an addiction. Furthermore, and relatedly, unlike those with addictions, particularly “unwilling” addicts, who clearly suffer from akrasia (weakness of will) in that they do not want to be addicted, Vaccine Refusers very much want to remain unvaccinated, they choose to do so, and they choose to do so willfully and freely.

This leads directly to the next section: Akrasia/Action/Agency/Will.

## Vaccine Refusal and Akrasia, Action, Agency, Will

### Akrasia, Action, Agency, Will

Akrasia (or weakness of will) has been pondered by philosophers at least as far back as Aristotle. The modern understanding is expressed by Donald Davidson [(33), pp. 21–42] who explains that the akrate is a person who acts on Y, even though he/she has judged that X is a better action (all things considered) than Y. A typical example finds Mr. O, who thinks he ought to give up smoking (Action X), nonetheless having a cigarette (Action Y), even after he's decided that not smoking that cigarette would be better than smoking it. In this case, Mr. O judges that Action X would be better than Action Y, and yet he smokes the cigarette, performing Action Y. Davidson [(33), p. 42] suggests that although the akratic person has *a* reason for acting in a manner against his/her best all-things-considered judgement, that this reason has not been supplanted by the *better* reason, renders the akrate not rational and moreover a person who “…cannot understand himself: he recognizes in his own internal behavior, something essentially absurd.”

The sort of rationality Davidson avers absent for the akratic person is the Philosophical/Psychological type. But in many akratic persons Biological Rationality would be violated too—there is not much selective fitness success gained by most substance addictions (and perhaps most social addictions too.) Yet and surprisingly, one could square akratic actions with an aspect of classical Economic Rationality—consistency. This would entail arguing that akratic people, while they do actually judge Action X better and yet perform Action Y, truly desire Action Y more than Action X, and perform the more desired, but akratic action, consistently.

Brakel [(34), pp. 150–151, 163–165]) suggests a possible remedy for “fixing” such desire-based akrasia. Take smoker, Mr. O. Suppose one juxtaposed O's desire for this particular cigarette now (Action Y_1_) against O's desire to be a non-smoker (Action X). By not smoking this particular cigarette, Mr. O performs Action X_1_. This can prove important if Action X_1_ can now be understood as the first of many such Actions—Action X_2_, Action X_3_, Action X_n_. By weighing the diachronic desire—to be a non-smoker—against each particular (synchronic-at-one-time) instance of desiring a single specific cigarette, the action judged as better, Action X, might have a chance.

Harry Frankfurt's work [(35), especially pp. 16–24] on agency and an agent's will, deepens this sort of understanding. Frankfurt outlines major differences between three types of addicts—“the unwilling addict,” the “wanton addict,” and the “willing addict.” That most addictions involve the user's initial wanting (and probably liking too) of the addictive entity, is Frankfurt's starting point. He terms this initial wanting “a first-order desire.” Important distinctions then begin. What is the attitude of the person who has become addicted to the entity of his/her first-order addictive desire? Does he/she desire to not have the desire for the substance, in other words, does he/she have a second-order desire to not be addicted? Further, is this second-order desire so important that the addict embraces this second-order desire as what he/she would intend to constitute as his/her will, and free agency? This attitude describes the “unwilling addict.” However, because of the strong pull of the first order desire, especially to various addictive substances, Frankfurt avers that the will of the “unwilling addict” can never be entirely free, notwithstanding the second-order volition to constitute him/herself as a non-addict. (p. 21). The “wanton addict” in contrast, has no particular second-order conative attitudes toward the addiction; he/she does not care what constitutes his/her agency or will. Thus, for Frankfurt, the “wanton addict” has no will at all (p. 21). Finally, we have the “willing addict” whose first- and second-order desires match, and who intends to embrace his/her addictive desires as constitutive of his/her actions, agency, and will, and even as part of his/her self-identity. But for Frankfurt (pp. 24–25) even this “willing addict” cannot have truly free will, “for his desire to take the drug will be effective regardless of whether or not he wants this desire to constitute his will.”

The Section titled “Is COVID-19 Vaccine Refusal any sort of Addiction?” concludes by holding that Vaccine Refusal, despite having some features in common with addiction, should not be classified as any sort of addiction. Now, in discussing akrasia and particularly the willing addict, there is a related, but more specific question: Should COVID-19 Vaccine Refusers be understood as demonstrating behaviors and taking actions that are akrasia-based? Again, the short answer is “no.” This is expanded upon in the next sections.

### Vaccine Refusal Is Not Due to Akrasia

Vaccine Refusal does not owe to akrasia. Indeed, Vaccine Refusers should be seen as frankly anti-akratic. This, insofar as it has become ever easier to get vaccinated, with fewer obvious material costs (including monetary ones) and it has become more problematic (and costly) to continue to refuse. First, the vaccines are readily available, increasingly so, and observable in many venues. Second, as more and more unvaccinated (vs. fully vaccinated) people become very sick, hospitalized, and die, the pandemic (at least in the UK and US) has become a pandemic of the unvaccinated. This information is far from hidden, in fact it is likely personally experienced by most, if not all. Thus, to the extent that Vaccine Refusers would also want to protect themselves and loved-ones, it is made harder to continue to refuse COVID-19 vaccinations, requiring more strength of intention and agential will. Finally, in areas where there are company-wide, city, state, or federal mandates for vaccinations, some Vaccine Refusers will lose their jobs and thus their incomes. Utilitarian reinforcement is largely on the side of getting the vaccine.

### Vaccine Refusal Is an Intentional Action of Free Will

The Vaccine Refuser endorses his/her refusal. Even if one were to insist on regarding Vaccine Refusal as a sort of addiction—perhaps an addiction to misinformation gathering, or an addictive thrall toward following a tribal group anti-vax leader—the Vaccine Refuser could only (and with many caveats) be considered a “willing addict.” Vaccine Refusers (at least the sort dealt with in this project) indeed want, in a second-order fashion too, their first-order desire—not-to-get-vaccinated. Further, they care about this desire and endorse it “whole-heartedly” (Frankfurt [(36), pp. 164–5]), embracing it as among the desires which they choose to constitute their will, agency, and self-identity. Frankfurt [(37), p. 111] puts the relations between second-order volitions, caring, and self-identity thus:

A person who cares about something is… invested in it. By caring about it, he makes himself susceptible to [its] benefits and vulnerable to [its] losses…[as] what he cares about flourishes or is diminished. We may say…in this sense he *identifies* [Frankfurt's emphasis] himself with what he cares about.

Whether or not one agrees with Frankfurt about the free will capacity of the “willing addict,”^16^ the powerful physiologic and almost automatic cravings associated with first-order wanting of addictive substances, do not apply for Vaccine Refusers. Thus, Vaccine Refusers—people who (a) choose in second-order fashion to make their first-order desire for Vaccine Refusal their will, and (b) choose Vaccine Refusal as an attitude and behavior to embrace, identifying with it wholeheartedly and unambivalently—are fully agential persons exercising their intentions with genuine free will.

## Primary Process/System One and Vaccine Refusal

### Everything Associated With Vaccine Refusal Leads to Increased Information Incentive

Association is the key here, as associational Primary Process/System One connections amplify the Vaccine Refusal stance. This occurs *via* increases in salient informational reinforcement, quite analogous to the phenomena of addicts' increased craving whenever in the presence of materials, persons, or contexts, associated with the addictive substances (28, 29). Obvious associational connections include belonging to the group (tribe) of politicians, media personalities, and celebrities who proudly proclaim their anti-COVID vax views, praising those who agree. More subtle are the COVID-related issues that are propounded by these same famous “influencers” and in social media connections, personal and public. These can include negative attitudes toward public health issues like masking (especially if there are mandates to mask), social distancing, high quality ventilation, quarantining when necessary, and crowd avoidance. More remote, but no less potent associational links, can find Vaccine Refusers harboring (and then even demonstrating) hostility toward public health officials, sometimes devolving into actual aggression toward doctors, nurses, and other health care workers. This stance too receiving tacit (if not actual) informational reinforcement salience *via* continued group approbation.

Associations can (and do) branch out from COVID-related matters to COVID-adjacent issues. Especially when advanced by the above-mentioned tribal group leaders (politicians, media and other famous persons), COVID-adjacent views can include anti-Asian sentiment since China is blamed for the pandemic, and anti-science protest and behavior.

Additional Primary Process/System One associational connections, farther away from the nidus of concern regarding COVID-19, also arise for Vaccine Refusers, much as they do for phobic psychiatric patients. For the latter case, what can begin as a fear of genital damage, can spread to a generalized hypochondria. For the Vaccine Refuser, refusing a COVID-19 injection can spread to refusing other vaccines, even those well tolerated in the past (37). Also, and equally far from the original core of COVID-19 Vaccine Refusal, anti-Asian prejudice can itself generalize first to anti-immigrant then anti-immigration bias—all *via* associative connections. The associational spread to matters no longer explicitly pandemic related at all can be seen too as Vaccine Refusers embrace right wing notions regarding “individual freedom.” These can be as specific as wanting lax (or no) gun regulations, and as general as demanding to be “free” from “elite” government regulation.

### Vaccine Refusal: Rigidity, Another System One/Primary Process Characteristic

Another characteristic of Primary Process/System One mentation can be observed in many COVID-19 Vaccine Refusers. Opposite from the seemingly free-wheeling associative connections discussed just above, a marked rigidity can be seen in Vaccine Refusers. Again, this Primary Process/System One insistence is quite like that seen both in early and middle phases of substance addiction^17^ and for certain psychiatric patients, for example those with simple phobias. While in terms of classic economic rationality, Vaccine Refusers and phobics of this type are not irrational in that they do not preference switch, there is something frankly irrational in not taking into account changing circumstances and/or contravening evidence. To illustrate, take (a) a dog phobic who was realistically afraid of an angry dog, but is trembling in the presence of a puppy or a guide dog and (b) a COVID-19 Vaccine Refuser who even in the face of increasing illnesses and deaths^18^ will not take a vaccine preventing a life-threatening outcome. Neither could meet anyone's idea of rationality. Timothy Williamson [38, p. 79] a British epistemologist puts it this way: “…profoundly dogmatic beliefs which are impervious to future evidence …may be even more likely to persist than beliefs rationally sensitive to future evidence that do constitute knowledge.”^19^

### System One/Primary Process Mentation Is Often Emotion Based

As was just presented above, it is hard to understand COVID-19 Vaccine Refusal as any kind of “rational” behavior. But consider this: Pleasures are best understood as primary process-mediated, a-rational phenomena—neither rational nor irrational—and Vaccine Refusal can bring refusers pleasure. This often takes the form of a sense of tribal identity, with warm feelings toward group members, praise from group leaders, and a sense of high-minded righteousness, all of which contribute to emotional satisfaction. Now along with the other sorts of elements outlined as informational reinforcers for Vaccine Refusal, one can add these tribal belonging-derived positive emotions. Given that System One/Primary Process mentation modes are more emotionally reactive and affect-close than those of System Two/Secondary Processes (15, 16, 18–20), one can also now better understand that tribal belonging identity and its related pleasures are not only salient but potent informational reinforcers, fueling continued COVID-19 vaccine refusal.

Loss aversion, another System One characteristic (18–20), is relevant here. While it might seem that compromising one's future health and perhaps one's chances for a long life would be considered a big loss, anything jeopardizing one's identity as a group member and the pleasure and satisfaction associated, is a more immediately *felt* loss, more emotional, and paradoxically bigger! The familiar System One/Primary Process operation of confirmation bias (18–20) functions here too. Guarding against what would be an immediate and serious emotional loss, evidential matters regarding COVID-19 vaccines as safe and effective are discounted or ignored, while any misinformation about vaccine dangers and COVID-19's harmlessness is sought out and promoted.^20^

## More On Vaccine Refusal and Group (Tribal) Identity

### Group Identity's Informational Reinforcement and a Positive Feedback Loop

Vaccine Refusers receive praise from tribe leaders for continued refusal. Their group identity is in this way reinforced. Moreover, the continued act of Vaccine Refusal is commended, by group leaders and fellow members, as representing the independent free choice of free agents exercising free will. Certainly, as was discussed earlier, the Vaccine Refusers of interest here, should be considered free agents with the free will to express and then act upon their intentions in refusing COVID-19 inoculations. Add to this, what amounts to a group cohesion marketing bonus: the free will choice of Vaccine Refusal is packaged as supporting the principles *Freedom, Independence, and Liberty*, writ large.

True, it is freedom *for individuals*, without much regard for *societal* freedoms. For a few examples: (a) there is little concern for others to be free *from* greater viral-spread from unvaccinated persons; (b) no consideration for hospital overload, and the freedoms interfered with, thereby, both to patients and health care workers; and (c) little understanding that by remaining unvaccinated, the pace of the pandemic's melioration is slowed, both in terms of economic recovery, and biologically as greater numbers of unvaccinated hosts provide an expanded and readily available source for viral proliferation and with this, variant evolution.

These societal problems in no way decrease Vaccine Refusal. The reverse is the case. Linking the action of refusing COVID-19 inoculations with Liberty, Freedom, and Independence provides a positive feedback loop, accelerating Vaccine Refusal. Disavowal of COVID-19 vaccinations is intensified, accentuating the already existing Primary Process-mediated rigidity. Group identity is reinforced; Vaccine Refusers now regard themselves as members a group embodying a set of worthy ideals. Some even consider Vaccine Refusal, and the attendant ideal of one's personal freedom, worth fighting for, perhaps even dying for. That this can be the case, entails some restructuring of one's SSR and LLR preference and reward system. We turn to that next.

### Alternative Structuring of SSRs and LLRs for Two Subgroups of Vaccine Refusers

In continuing with the effort to explore Vaccine Refusal, we have found it useful to divide the Vaccine Refusal cohort under exploration into two different subgroups:

Subgroup-A—Those who refuse COVID-19 vaccines, believing that COVID-19 vaccines DO NOT facilitate long term better health; some in this subgroup actually holding instead that the vaccines have more deleterious health side effects (long and short term) than COVID-19.

Subgroup-B—Those who refuse to be vaccinated despite believing that the COVID-19 vaccines DO improve survival rates, diminish serious illness, and thereby allow better health in the long range future.

Clearly the structuring of the reward preferences for these two groups will be different, with Subgroup-B having to make some compromises in order to persist in Vaccine Refusal. Below is a simple chart comparing the two subgroups.

**Subgroup-A: Belief That Vaccines DO NOT Promote Future Better Health**

**If take vaccine**

**SSR**                                       **LLR**

**Negative—Against Tribe     Zero—No LLR**

**Negative—Possible Short-term and/or Long-term Side**

**Effects**

                              *************

**If refuse vaccine**

**Positive—With Tribe             Positive—With Tribe**

**Positive—Individual             Positive—Individual**

**Freedom                                Freedom**

**Positive—Avoiding Possible Short-term and Long-term**

**Side Effects**

**Subgroup-B: Belief That Vaccines DO Promote Future Better Health**

**If take vaccine**

**SSR**                                       **LLR**

**Negative—Against Tribe     Good Long-term**

**Heath**

                              *************

**If refuse vaccine**

**Positive—With Tribe             Positive—Positive—With Tribe**

**Positive—Individual             Positive—Individual**

**Freedom                                Freedom**

**Negative—If Sick, especially Very Sick**

Even from these very rudimentary comparison charts, one can see that for persons in Subgroup-A, Vaccine Refusal gains “positives” both in terms of Short-term Small rewards and Long-term Large rewards. Partly this owes to Subgroup-A's responsiveness mostly to informational reinforcement. Indeed, for those in Subgroup-A, what should function as the utilitarian incentives of better health, are disregarded, and even distorted as the vaccine is regarded as more dangerous than the virus. Those in Subgroup-B, on the other hand, must deal with conflicts in structuring both their anticipated SSRs and LLRs as they choose (and continue to choose) COVID-19 Vaccine Refusal. Partly this is the case because both utilitarian and informational reinforcements are salient and rewarding for those in Subgroup-B.

More will follow about each of these Subgroups in the next section on matters of epistemology.

## Epistemological Issues

Continuing with the Subgroups described just above, Subgroup-B—those who do believe that COVID-19 vaccines are protective of future good health—is the subgroup that can be more readily explained epistemologically. Thus, people in this subgroup of Vaccine Refusers believe the evidence about vaccine efficacy and safety, and thus know that they are discounting the future, decreasing their LLRs. Nonetheless, they choose to remain as Vaccine Refusers since the combination of positive informational reinforcement SSRs and LLRs gained by tribal identity and individual freedom outweighs the big utilitarian negative of possibly getting severely ill, hospitalized, and dying.

Representing this Subgroup symbolically:

**Persons in Subgroup-B:**

**Know X, where X** **=** **facts about the vaccine;**

**so they also Know Y**,

**where Y** **=** **no vaccine →** **decreased LLR**

Subgroup-B persons are squarely in the category of the non-akratic free willed agents discussed at length above. They are like the “Willing Addicts” but with truly free choice, in that their primary desire (to be unvaccinated) does not in itself have the powerful physiologic compulsive drive common to many addictive substances.

Now, turn to the Vaccine Refusers in Subgroup-A. Those in this group choose to refuse vaccination with less conflict. They do not believe the evidence that the available vaccines safely promote protection from severe COVID-19 morbidity and mortality; this long-term large utilitarian reinforcement does not count as reward. Thus, people in this subgroup do not know that they are discounting the future and diminishing their LLRs with respect to improved long-term health. Instead, they chose actions reinforced by informational reinforcement rewards, long and large, small and short, all linked to putative individual freedom and actual tribe identity cohesion.

But, behold: the phenomenon of “not-knowing” admits of two different types, each important to explore. So, dividing Subgroup-A further, Subgroup-A-1 and Subgroup-A-2 arise. Those in Subgroup-A-1 actually *do not know* the essential accepted (well-known) facts about COVID-19, and COVID-19 vaccines. Briefly, these facts are: COVID-19 can be a life-threatening illness, the most severe consequences of which can be prevented and future good health promoted, by taking a COVID-19 vaccine (1). Turning to Subgroup-A-2, these not-knowers are different. Instead of believing the evidence that COVID-19 vaccines are safe, effective, and thereby enhancing to future good health, they “know” the following: COVID-19 vaccines are neither safe nor effective; the vaccines cause more long-term health damage than COVID-19; and COVID-19 is not a dangerous infectious disease. People in Subgroup-A-2 can, in this fashion, readily conclude that there is no discounting of the future in Vaccine Refusal, as there is no long-term large reward (LLR) of any salience in taking the vaccine.

Both of these not-knowing subgroups are interesting from an epistemological view. Representing them symbolically:

**Persons in Subgroup-A-1:**

**do NOT-know X (vaccine facts), so they also**

**do NOT-know Y (if vaccine refused →** **decreased LLR)**

Of great interest about this group, is that X (and Y which follows from X) is there to be known, everywhere; certainly, avoiding true facts about the virus and its vaccines must take a considerable amount of work. Indeed, following from the understanding of PP-Rationality above, this group seems to be irrational.

**Persons in Subgroup-A-2:**

**“Know” NOT-X (vaccine falsehoods) so they also**

**“Know” NOT-Y (vaccines do not improve LLR)**

“Know” appears in quotes in that one cannot know something that is not true.^21^ Technically we might instead indicate that persons in Subgroup-A-2 believe NOT-X (and the NOT-Y that follows), i.e., they have false beliefs.

Now, looking at Subgroups-A-1 and A-2 together, a vexing epistemological problem is revealed: Is there a relation between **NOT-Knowing X** and **“Knowing” (believing) NOT-X to be true?** What is the relation? How are they similar; how are they different?

A workable solution can be found in Williamson's (38) seminal book, *Knowledge and Its Limits*. In this work, Williamson holds that knowledge is prior to belief, advancing a view radically at odds with the conventional position that knowledge is constituted by justified true belief. Williamson states (p. 47): “To know is not merely to believe while various other conditions are met; it is to be in a new kind of state, a factive state.” As such, instead of knowledge consisting of belief rendered true by evidence, Williamson (p. 47) argues for the reverse: In order for beliefs to be held, and held as true, there has to be prior knowledge, with the knowledge constituting evidence for the belief. “Knowledge sets the standard of appropriateness of belief…belief aims at knowledge (not just truth)…knowledge is the evidential standard for the justification of belief.” Moreover, and with clear relevance to this article's project, Williamson (p. 15) concludes that “…rationality requires one to conform one's beliefs to one's evidence… [i.e., to one's knowledge]. ”

To put these in a series:

(1) Knowledge = Evidence;
(2) Evidence from Knowledge is Necessary for (True) Beliefs;
(3) Rationality requires conforming one's beliefs to one's
(4) evidence/knowledge.

But, as is widely held and expressed also by Williamson (p. 14): “Granted that knowing is a mental state, one should therefore not be surprised that one can fail to know something without being in a position to know that one fails to know.”

With some frequency then, people don't know that they don't know; they don't know that they don't have the evidence for something they falsely believe they know. Often, instead, they believe (falsely) that they know (have evidence for) something else, asserting that they “know” this something else. That is the case for Vaccine Refusers in Subgroup-A-2. They claim they “know” (and have evidence) that COVID-19 vaccines are not protective and indeed harmful; and that they “know” (and have evidence) that the proposition: “No COVID-19 vaccine → decreased LLR” is a false proposition. They claim they “know” NOT-X (vaccine falsehoods) and likewise “know” the NOT-Y (vaccines do not improve LLR) which follows. Since NOT-X is false, Subgroup-A-2's Vaccine Refusal is based on a series of false beliefs that COVID-19 vaccines are not protective, but harmful, while the COVID-19 virus is not potentially severe, long-lasting, and too often life-threatening.

The Vaccine Refusers in Subgroup-A-1 are different. If they really do NOT-Know X, where X represents the actual facts about COVID-19 and the vaccines protecting against serious outcomes,^22^ and to the extent that there are no claims of “knowledge” of some NOT-X, i.e., incorrect assertions about COVID-19 and the COVID-vaccines mitigating severe disease; then this group of Vaccine Refusers has no basis for Vaccine Refusal beliefs—these are beliefs without any evidential grounds (no K, no E), and as such these beliefs are not merely false, but empty.

## Conclusions

The Vaccine Refusers explored in this project, do discount the future in favor of short-term rewards. But, unlike substance addicts, Vaccine Refusers are not akratic; they are instead, very firm in the resolve entailed by refusing vaccination. Further, unlike substance addicts these Vaccine Refusers display truly free will, which they exercise with full agential intentions, as they choose to refuse COVID-19 vaccination. They are driven more by emotions consequent to informationally salient reinforcement than by the rewards occasioned by utilitarian reinforcers. The informational reinforcement is complex—praise from group members and leaders, the promise of cohesive group membership, ideals propounded by the group that importantly include the principles of Individual Freedom and Liberty as instantiated by continued Vaccine Refusal. This last constitutes a positive feedback loop hardening the resolve of the Vaccine Refuser to continue his/her “independent” action for freedom.

Vaccine Refusals can be considered rational actions, at least in terms of two of the three types of rationality we have discussed. Many COVID-19 Vaccine Refusers do match their beliefs to their evidence, faulty though their evidence might be—fulfilling one criterion of Psychological/Philosophical Rationality. And in terms of Economic Rationality, there is almost zero preference switching, something seen routinely in stages of substance addiction. Biological Rationality, on the other hand, cannot accommodate Vaccine Refusal. There is no way to construe eschewing a health and life-promoting preventative treatment as advancing selective reproductive (or even extended inclusive) fitness.

Vaccine Refusal also demonstrates much that should be considered Primary Process/System One a-rationality: (a) Vaccine refusal spreads in an associative chain to issues both COVID-related and beyond; (b) there is an a-rational rigidity in the Vaccine Refusal behavior; and (c) decisions involving Vaccine Refusal are often reactive and emotion-based. Certainly, although it is the case that a-rationality is a mode of mentation that can be adaptive—many non-human animals operate with this sort of mentation as basic—a-rationality can be in the service of irrational ends too. As such it is difficult to regard Vaccine Refusal in the current context as anything but largely irrational.

Relatedly, from the epistemological view that knowledge is the source for evidence and that evidence is needed for belief, Vaccine Refusal entails two sorts of epistemological problems. One type of Vaccine Refuser refuses on the basis of rejecting available evidence (knowledge), instead, forming false beliefs on the basis of wrong evidence. The false beliefs are taken as knowledge, as is the wrong evidence. Another type of Vaccine Refuser refuses, claiming to not know the relevant, highly available facts (evidence) about COVID-19 diseases and the COVID-19 vaccines preventing major harm. This second sort of Vaccine Refuser acts on beliefs devoid of evidential content—their beliefs are empty more than wrong.

There is the possibility that in discovering these two types of Vaccine Refusal knowledge failures—false belief “knowing NOT-X” and empty belief “Not knowing-X”—we have advanced epistemological understanding just a bit. Similarly, in addressing the phenomenon of COVID-19 Vaccine Refusal—a singular and striking phenomenon, given the context of the current pandemic times—we might have added something to work (a) in the philosophy of action (addiction, akrasia, free will); (b) on the economic topics of instrumental vs. utilitarian reinforcement; and finally (c) dual processes, rational/a-rational/irrational aspects of psychology. However, whether or not our project has been helpful to these very well-established disciplines, we do feel we have provided a deeper, more overarching interdisciplinary understanding of COVID-19 Vaccine Refusal in the times of COVID-19, and while that is reward enough for us,^23^ policy-makers might appreciate a more direct indication of how this work might be of practical use.

Thus, recognizing that Vaccine Refusal is an agential free choice that is nonetheless System One/Primary Process mediated and fueled by largely instrumental reinforcement could aid in the design of programs tailored to these features. For example, suppose that getting COVID-19 vaccines were publicized as volunteering to join “The World-Wide Virus War.” This would be a vaccination campaign emphasizing Freedom—one's personal freedom, and freedom from the virus, marked by a military style tribal belonging symbol given with the initial jab. The symbolic item —not unlike the Trump MAGA hats—would have some sort of insignia to which each additional vaccine and booster would be registered with an extra bar or stripe added.

Indeed, such a program would have, and likely would have had, a better chance for success were it not for the ongoing weaponizing of (what should have been neutral) information about the dangers of the COVID-19 virus and the successes of the COVID-19 vaccines. Here “The War” is framed as that between Vaccine Refuser freedom-loving soldiers vs. Vaccine Believers who would deny your privacy and personal freedom. The weaponizing of information relates to another matter addressed in this article—the dual epistemological problems of empty belief and false belief. These manifestations of both mis- and dis-information are problems of pandemic proportion at present. One hopes that these dangers too can be minimized (becoming “merely endemic”) in a case-by-case manner; this through interdisciplinary investigations of the multiple and complex underlying factors, such as those outlined here regarding COVID-19 Vaccine Refusal.

## Data Availability Statement

The original contributions presented in the study are included in the article/supplementary material. Further inquiries can be directed to the corresponding author.

## Author Contributions

All authors listed have made a substantial, direct, and intellectual contribution to the work and approved it for publication.

## Conflict of Interest

The authors declare that the research was conducted in the absence of any commercial or financial relationships that could be construed as a potential conflict of interest.

## Publisher's Note

All claims expressed in this article are solely those of the authors and do not necessarily represent those of their affiliated organizations, or those of the publisher, the editors and the reviewers. Any product that may be evaluated in this article, or claim that may be made by its manufacturer, is not guaranteed or endorsed by the publisher.

## Footnotes

^1^For this article only “COVID-19 Vaccine Refusers” are considered, not people who are best characterized as vaccine-hesitant about COVID-19 vaccination.

^2^A recent New York Times article (2) (February 1, 2022), for example, supplied an easy-to-grasp graphic showing contrasts among deaths per 100,000 people from October to November 2021. Here the unvaccinated had 7.8 deaths, the vaccinated with no third shot (booster) had 0.6 deaths, and the thrice vaccinated had 0.1. No need for any statistics training to understand the significance of these differences.

^3^In various different writings from 1893 to 1896 Freud offered earlier, but less elaborated presentations. [Although tangential to the current work, for those interested these include Freud (1895), pp. 324–327, pp. 334–340, p. 357, p. 362; Freud (1893–1895), p. 9, p. 15, pp. 208–209, p, 214, p. 225, p. 239; and Freud (1896), pp. 198–199].

^4^Although there is a clear contrast between Primary and Secondary Process, these almost never operate in pure culture. Rather, most human mentation consists of a blend, with conflicts and compromises, in which one or the other type of mentation predominates.

^5^Like the Primary and Secondary Processes, Systems One and Two co-exist, sometimes in conflict, sometimes collaborating, often one or the other predominating. It is for heuristic purposes that they are discussed as operating singularly.

^6^The particular example, increased group cohesion, can have a down-stream result—enhanced selective reproductive success, a clear evolutionary fitness advantage. For more on this and other System One evolutionary advantages, see Gigerenzer and Todd (21); Todd and Gigerenzer (22).

^7^Indeed, over the decades the American Psychiatric Society's DSM (Diagnostic and Statistics Manual of Mental Disorders) has made many definitional changes. According to DSM-5 (23), the most recent version, there are 11 criteria for a Substance Use or Addictive Disorder. (Addictions other than those to substances—e.g., gambling, compulsive shopping, kleptomania—are still under some debate.) The 11 are: Hazardous use, social problems owing to use, neglect of functional roles because of use, withdrawal effects, tolerance (requiring more substance), using more substance, spending more time using, failed attempts to control, failed attempts to give up use, physical or psychological problems directly pertaining to use, cravings. To meet criteria, one must have two or more of these criteria. With 2–3 criteria, the disorder is considered mild, 4–5 moderate, 6 or more constitutes severe Substance Abuse Disorder.

^8^More will be said below on liking vs. wanting, largely in terms of brain circuitry.

^9^For example, according to the CDC website (1)“All currently approved or authorized COVID-19 vaccines are *safe* and *effective* and *reduce your risk* of severe illness. (CDC's emphasis)”.

^10^In the US, these include the mRNA vaccines Moderna and Pfizer, and the viral vector Johnson and Johnson; in the UK, the above mentioned three as well as the Astra-Zeneca's viral vector vaccine, and Novavax, a vaccine of the protein subunit type.

^11^A small number of cases of myocarditis have been attributed to side effects from the mRNA vaccines. These have been reported mostly in male adolescents and young adult men. Viral vector injections have been implicated in a rare and unusual blood clotting problem, mostly in young to middle-aged woman.

^12^Note that in employing the word “reinforcement” it is suggested that behavioral conditioning is an important part of consumer behavior, including addictive consuming. However, we do not have a reductive behaviorist view. This has been signaled by indicating that reinforcements “contribute” to behavior, and will become even clearer as our broad view of what constitutes “reinforcements” will be demonstrated in section “Vaccine Refusal and Akrasia, Action, Agency, Will” on Akrasia, Action, Agency, Will.

^13^The importance of associational connections as described above in the description of System One/ Primary Process operations will be taken up more fully in Section “Primary Process/System One and Vaccine Refusal” below.

^14^Here, we refer to “unwilling addicts,” persons who want to be free of their addiction. The “willing addict” will be examined in a later section. As will be clear then, these distinctions and their definitions [which owe to Frankfurt (30)] are important for the view of Vaccine Refusers advanced.

^15^It would be interesting to see comparative studies on the liking vs. wanting circuits in Vaccine Refusers vs Substance Addicts. If our hypothesis is correct, the Vaccine Refusers' brains will not show the Substance Addict's pattern of differential amplification of the wanting circuits over those that mediate liking.

^16^Indeed, the authors do not agree, although the argument against Frankfurt's view, is an issue outside the current matters of concern.

^17^Foxall [(7), p.144] characterizes addiction as the persistent demand/consumption of a commodity even when the cost increases sharply.

^18^Also, increased societal pressures, including possible personal job loss, as well as overwhelmed hospitals, and damaged economy.

^19^More will be said about Williamson's views on belief, evidence, and knowledge in Section “Epistemological Issues”.

^20^Is confirmation bias a species of what Foxall [(7), pp. 140–141] terms “reflective impulsivity,” i.e., a failure to accrue sufficient evidence before acting? This is something to be addressed in Section “Epistemological Issues”, as the view that evidence (from knowledge) is necessary for beliefs, is explored.

^21^According to Williamson [(38), p.21], “Knowing…is a *factive* attitude; one knows *p* if *p* is true, although one can be acquainted with the proposition *p* even if it is false.” [Williamson's emphasis].

^22^Although it seems hard to countenance, and increasingly so, there may indeed be persons who remain truly without knowledge about COVID-19 and COVID-19 vaccines. Again, those in this subgroup whose ignorance is purposeful and willful might be properly considered Philosophically/Psychologically irrational.

^23^While making this statement confidently, the authors are aware that the theme has not been exhausted. There is more to be said in the context of both PP- and B-rationalities, both of which impinge on E-Rationality. See, for instance, Okasha (39).
